# Ubiquitination regulation of mitochondrial homeostasis: a new sight for the treatment of gastrointestinal tumors

**DOI:** 10.3389/fimmu.2025.1533007

**Published:** 2025-03-11

**Authors:** Bingqian Huang, Yulin Yang, Jinming Liu, Biao Zhang, Nengming Lin

**Affiliations:** ^1^ Key Laboratory of Clinical Cancer Pharmacology and Toxicology Research of Zhejiang Province, Affiliated Hangzhou First People’s Hospital, School of Medicine, Westlake University, Hangzhou, China; ^2^ School of Clinical Chinese Medicine, Gansu University of Chinese Medicine, Gansu, China; ^3^ Department of General Surgery, The First Affiliated Hospital of Dalian Medical University, Dalian, China

**Keywords:** mitochondrial homeostasis, ubiquitination, gastrointestinal tumors, mitochondrial biogenesis, mitochondrial dynamics, mitophagy, mitochondrial metabolism

## Abstract

Mitochondrial homeostasis (MH) refers to the dynamic balance of mitochondrial number, function, and quality within cells. Maintaining MH is significant in the occurrence, development, and clinical treatment of Gastrointestinal (GI) tumors. Ubiquitination, as an important post-translational modification mechanism of proteins, plays a central role in the regulation of MH. Over the past decade, research on the regulation of MH by ubiquitination has focused on mitochondrial biogenesis, mitochondrial dynamics, Mitophagy, and mitochondrial metabolism during these processes. This review summarizes the mechanism and potential therapeutic targets of ubiquitin (Ub)-regulated MH intervention in GI tumors.

## 1 Introduction

Gastrointestinal (GI) tumors, including hepatocellular carcinoma (HCC), esophageal cancer (ESCA), gastric cancer (GC), colorectal cancer (CRC), and pancreatic cancer (PAAD) (1), are the leading causes of cancer-related deaths worldwide. Highlighting its severe public health burden, there are an estimated 4.9 million new cases and 3.9 million deaths annually, according to the latest data (2). Mitochondria (Mt) plays a crucial role in the occurrence and development of GI tumors (3). As a key organelle in the metabolic reprogramming of cancer cells, Mt dysfunction is one of the main drivers of cancer initiation and progression (4). Mt stress releases Mitochondrial DNA (mtDNA) into the cytoplasm and extracellular space, activating multiple innate immune signals (5). In GI tumors, the mtDNA mutation rate is higher, mainly in the D-loop region, which is the hypervariable region of mtDNA and is responsible for the regulation of mtDNA transcription and replication, which may be related to the special physiological environment of gastrointestinal cells (such as acidic environment, frequent cell turnover, etc.) (6, 7). Concurrently, the increased electron leakage from Mt stress generates a high concentration of reactive oxygen species (ROS), which further aggravates tissue damage and inflammation and suppresses the signal presentation between dendritic cells and T cells, leading to immune cell dysfunction and further promoting the infiltration of tumor-associated macrophages and the formation of an immunosuppressive tumor microenvironment (8, 9). Additionally, DNA and ROS released by Mt can change the balance of intestinal microbiota, leading to damage to the intestinal barrier and the occurrence of inflammatory bowel disease (IBD) (10). IBD is one of the risk factors for CRC (11). In the late GI tumors stage, Mitochondrial metabolism (MM) may instead increase, promoting cancer growth (10). This may be related to the B-cell lymphoma/leukemia-2 (BCL-2) protein family promoting the increase of mitochondrial permeability transition and mitochondrial permeability transition resistance, affecting the release of cytochrome C, thereby promoting the malignant transformation and progression of tumors (12). Studies have shown that Mt can also act as strategic molecular intermediaries or transport media for targeted therapies involved in antitumor activities (13). In summary, Mt dysfunction and structural damage further complicate the mechanism of GI tumorigenesis and development. Therefore, maintaining the normal function of Mt and regulating Mitochondrial homeostasis (MH) is key to intervening in the progression of GI tumors. MH refers to a state of balance in which Mt maintains normal function and structure within cells, including processes such as Mitochondrial biogenesis (MB), Mitochondrial dynamics (MK), Mitophagy, and MM (14–16).

**Figure 1:**
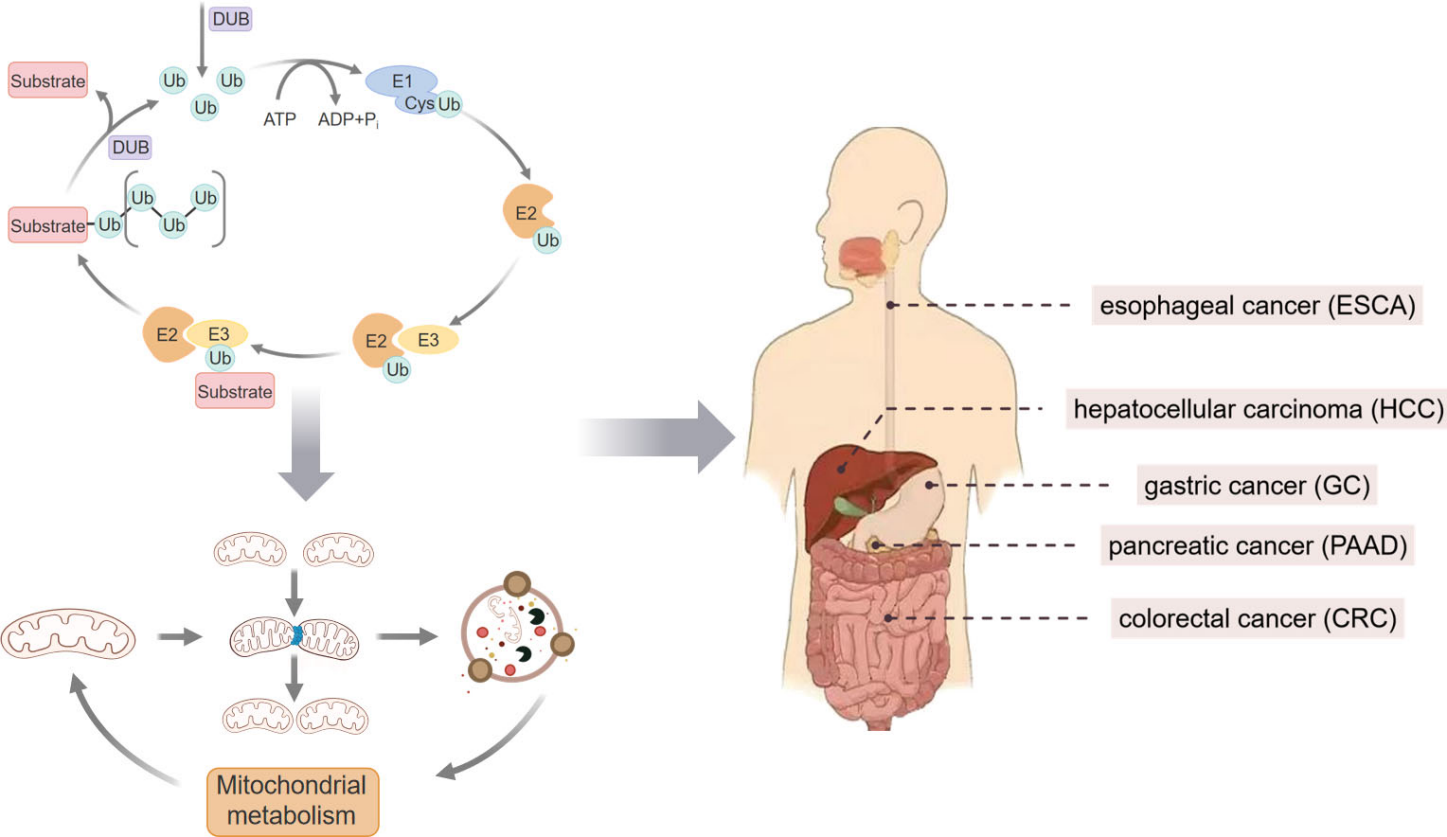
Schematic representation of MH targeted by ubiquitination affecting GI tumors.

Ubiquitination is involved in regulating these processes to maintain normal cellular functions. Ubiquitination is one of the main post-translational modifications of proteins, involving the addition of Ub molecules to target proteins, and plays a role in regulating protein degradation, signal transduction, DNA repair, cell cycle, and apoptosis within organisms (17–19). The process of ubiquitination is primarily catalyzed by E1 Ub-activating enzymes, E2 Ub-conjugating enzymes, and E3 Ub ligases, which play roles at different stages of the enzymatic ubiquitination cascade. Deubiquitinating enzymes (DUBs) can reverse ubiquitination by removing Ub. Under normal conditions, the ubiquitination process balances MH through the action of ubiquitinating enzymes and DUBs (20). However, when the ubiquitination process is disrupted, and MH is imbalanced, the progression of GI tumors is affected (**Figure 1**). This study to elucidate the molecular mechanisms and therapeutic targets of ubiquitination-regulated MH in the intervention of gastrointestinal tumors by reviewing the literature on Mt, ubiquitination, and GI tumors published in the past decade from databases such as PubMed and Web of Science, thereby providing theoretical support for the clinical diagnosis and treatment of GI tumors.

## 2 Ubiquitination regulates MH through multiple pathways

### 2.1 Ubiquitination targeting MB

MB refers to the synthesis of new Mt within cells, a process activated by various physiological and environmental signals such as cellular stress, increased energy demands, exercise training, and hormonal changes (21). This process primarily involves the transcriptional activation of nuclear-encoded mitochondrial genes, the translocation of corresponding proteins to Mt, the replication of mtDNA, and the synthesis of mitochondrial phospholipids (22). We found that ubiquitination targets peroxisome proliferator-activated receptor gamma coactivator-1 alpha (PGC-1α) to participate in the transcriptional activation of nuclear-encoded mitochondrial genes, Mitochondrial ubiquitin ligase/the E3 ligase Membrane-associated ring-gh-type finger 5 (MITOL/MARCH5) ubiquitinates DNA polymerase gamma catalytic subunit (PolγA) to mediate mtDNA replication, and the tumor necrosis factor receptor-associated aactor 6 (TRAF6) E3 ligase restricts protein translocation, while mitochondrial phospholipid synthesis and membrane changes also reciprocally affect ubiquitination (23, 24).

#### 2.1.1 Regulation of PGC-1α

PGC-1α is a primary regulatory factor in MB and energy metabolism, controlling both the nuclear and mitochondrial genomes (25, 26). PGC-1α regulates mtDNA replication, transcription, and translation, as well as the assembly of oxidative phosphorylation (OXPHOS), through co-activating nuclear respiratory factors (NRFs) and estrogen-related receptor alpha, thus maintaining mitochondrial quantity and function (27). Additionally, as a cellular energy responder, PGC-1α is activated via the AMP-activated protein kinase/silent information regulator 1 (SIRT1) pathway, enhancing MB (28). The heme oxygenase 1/PGC-1α pathway responds to oxidative stress by directly activating antioxidant enzymes such as superoxide dismutase, catalase, and glutathione peroxidase, thereby improving the cell’s antioxidant capacity, reducing ROS production, maintaining mitochondrial adenosine-triphosphate (ATP) levels and membrane potential (MMP), and protecting Mt from oxidative stress-induced damage (29).

The mechanisms of ubiquitination regulating PGC-1α mainly include promoting PGC-1α degradation, modulating its stability, and controlling its activity. Research has shown that radiation-induced DNA-dependent protein kinase can phosphorylate serine 636 of PGC-1α, thereby enhancing the binding of the E3 Ub ligase Ring finger protein 34 to PGC-1α and accelerating its ubiquitination and degradation (25). However, this binding can be competitively blocked by lysine methyltransferase 5C, which reduces the ubiquitination level of PGC-1α and extends its half-life (30). In addition, neural precursor cell expressed, developmentally downregulated protein 4-1 (NEDD4-1) is also an E3 ligase that can mediate the ubiquitination and degradation of PGC-1α by enhancing the recognition of the “TPPTTPP” sequence in PGC-1α through phosphorylation mediated by glycogen synthase kinase 3β (GSK-3β) (31).

#### 2.1.2 MITOL ubiquitinates PolγA

The mitochondrial membrane-integrated Ub ligase MITOL is a key regulator of mitochondrial membrane fission, fusion, and mitophagy (32). PolγA is the only DNA polymerase in Mt, and it replicates mtDNA through a process known as “D-loop replication,” where the heavy strand is replicated first, followed by the light strand (33). Recent studies have shown that MITOL ubiquitinates PolγA, negatively regulating its interaction with the translocase of the outer mitochondrial membrane 20 (Tom20), thereby inhibiting its entry into Mt (34).

#### 2.1.3 TRAF6 restricts p53

TRAF6 limits the translocation of tumor protein 53(p53) toMt by promoting the ubiquitination of p53 at lysine 24 (K24) in the cytoplasm through K63-linked Ub chains. This modification restricts the interaction of p53 with myeloid cell leukemia-1 (MCL-1)/BCL-2 Antagonist 1 (BAK), thereby inhibiting p53 mitochondrial translocation (35).

#### 2.1.4 Ub ligases regulating mitochondrial phospholipid synthesis

Cardiolipin (CL) is a characteristic phospholipid of the mitochondrial inner membrane, playing a crucial role in cellular energy metabolism, MK, and the initiation of apoptotic pathways (36). The precursor for CL synthesis is phosphatidic acid (PA), which is synthesized in the endoplasmic reticulum and then transported to the outer mitochondrial membrane (OMM) (37). From there, it is transferred to the inner membrane for CL synthesis by the uncharacterized protein with a phosphatidic acid transfer protein-like domain1-mitochondrial distribution and morphology Protein 35 (MDM35) protein complex in the intermembrane space (38). Studies have shown that common lipids in the mitochondrial membrane interact with MITOL and influence its activity and stability depending on the *in vitro* lipid composition (39). Notably, the binding of CL to purified MITOL significantly reduces its thermal stability, whereas the presence of PA enhances its stability most strongly (32), which further confirms that lipids can directly affect the activity of Ub ligases and may control the ubiquitination-dependent mechanisms regulating MK and turnover. Phosphatidylcholine is a lipid essential for mitochondrial membrane construction, primarily synthesized through the Kennedy pathway using choline as a substrate. During phosphatidylcholine synthesis, choline kinase accelerates mitochondrial damage. The mitochondrial kinase PTEN-induced putative kinase 1 (PINK1) accumulates on the membrane of damaged Mt, activates the Parkin rbr E3 ub protein Ligase (Parkin), and promotes substrate ubiquitination to initiate mitophagy (40).

### 2.2 Ubiquitination regulates MK

Mt are highly dynamic organelles capable of undergoing continuous cycles of fusion and fission, thereby altering their morphology, size, and spatial distribution (41). This physiological process is referred to as MK. MK refers to the state in which Mt maintains homeostasis through fusion and fission processes in cells. This dynamic change causes Mt to assume a variety of morphologies in the cytoplasm, such as punctate, fragmented, strip, or linear. MK is closely related to the functions of Mt, such as cell proliferation, cell metabolism, and cell migration (42). This balance is maintained by various dynamin-related guanosine triphosphatase (GTPases), which play critical roles in these processes (43). Key proteins involved include dynamin-related protein 1 (Drp1), mitofusin 1 (MFN1), mitofusin 2 (MFN2), and aptic atrophy 1 (OPA1), which mediate membrane dynamics and structural changes (44).

#### 2.2.1 The recruitment of Drp1

Drp1 is an essential protein in the mitochondrial fission process, containing a GTPase domain that enables it to bind and hydrolyze GTP (45). Drp1 primarily resides in the cytoplasm and, in response to mitochondrial fission signals, is recruited to the mitochondrial surface. There, it assembles into ring-like structures and utilizes the energy from GTP hydrolysis to divide Mt into two separate units (46). Identified Drp1 receptors include mitochondrial fission factor (Fis1), mitochondrial fission factor, and MK proteins of 49 kDa and 51 kDa (47). These receptor proteins facilitate the localization of Drp1 toMt through protein-protein interactions. Recent studies have revealed that the clustered Mt homolog gene and its drosophila homolog clueless promote the recruitment of Drp1 to the mitochondrial surface via its receptors in both human cell lines and Drosophila models, thereby enhancing mitochondrial fission (48). Current studies have shown that Drp1 can be targeted for ubiquitination and degradation by E3 Ub ligases, such as MITOL and Parkin, thereby influencing the normal mitochondrial fission-fusion process (49, 50). Knockout of either enzyme results in increased Drp1 activity and uncontrolled mitochondrial hyper-fragmentation (49). In cells lacking MITOL, re-expression of MITOL reverses the suppression of stress-induced apoptosis (51). Furthermore, emerging evidence indicates that Drp1 is not only a substrate of MITOL but also a regulatory factor of MITOL activity. Drp1 may modulate MK by influencing the functional activity of MITOL (52).

#### 2.2.2 MFN1 and MFN2

The mitochondrial fusion proteins MFN1, MFN2, and OPA1 are essential GTPases responsible for the structural fusion of mitochondrial membranes. MFN1 and MFN2 regulate the fusion of the OMM, while OPA1 mediates the fusion of the inner mitochondrial membrane (IMM) (53). Studies have shown that a family with sequence similarity 73 member A/B promotes mitochondrial fusion through the regulation of phospholipid metabolism, particularly via mitochondrial phospholipase D, in collaboration with MFN1/2 on the OMM (54). Additionally, the active domain of G-protein β2 (Gβ2) undergoes structural remodeling with MFN1’s binding domain, regulating MFN1 migration on the mitochondrial membrane and facilitating mitochondrial fusion. However, Gβ2 does not interact with MFN2 (55). Sudeshna Nag et al. (56) reported that under stress conditions induced by carbonyl cyanide 3-chlorophenylhydrazone (CCCP), the interaction between mitochondrial phosphatase phosphoglycerate mutase 5 (PGAM5) and MFN2 is weakened. Instead, PGAM5 shifts to interact with Drp1. Concurrently, MFN2 undergoes phosphorylation and ubiquitination by kinases and E3 Ub ligases, leading to proteasomal degradation. This results in an increased proportion of Mt failing to undergo fusion, as well as mitochondrial fragmentation and degradation. This process can be reversed by deubiquitinating enzymes such as Ub-specific protease 30 (USP30). USP30, a deubiquitinase, inhibits mitochondrial fusion by reducing the non-degradative ubiquitination levels of MFN1/2 (57). The SCFMdm30 complex, an intracellular E3 Ub ligase complex composed of S phase kinase-associated protein 1, Cullin1 (CUL1), F-box protein (FBX), and Ring-box 1, promotes the K48-linked ubiquitination of fusion of Mt protein 1 (Fzo1) (the yeast homolog of MFN1/2), leading to its degradation and subsequently impairing mitochondrial fusion (58). In contrast, USP2 specifically binds to the ubiquitinated form of Fzo1, removing the Ub modification to regulate Fzo1 stability and enhance mitochondrial fusion efficiency (59).

#### 2.2.3 OPA1

Compared to MFN1/2, OPA1 exhibits a broader range of functions, including maintaining the respiratory chain, MMP, cristae structure, regulation of apoptosis, and mitochondrial DNA stability (60). OPA1 exists in multiple isoforms, primarily categorized into long isoforms (L-OPA1, including a and b) and short isoforms (S-OPA1, including c, d, and e). L-OPA1 predominantly regulates the fusion of IMM, while S-OPA1 is involved in IMM fission (61). Studies have shown that treatment of SH-SY5Y cells with 6-hydroxydopamine (6-OHDA) results in a decrease in the protein levels of MFN2 and OPA1. Conversely, 6-OHDA treatment increases the expression of Fis1 and Drp1, leading to excessive mitochondrial fission, thereby affecting mitochondrial morphology and function (62). Moreover, the study found that carnosic acid, a rosemary extract, enhances the ubiquitination of inhibitor of nuclear factor kappa-B kinase subunit gamma (IKKγ), activating the Parkin/IKKγ/p65 signaling pathway to upregulate OPA1 expression and maintain MK homeostasis (62). Optic atrophy 1 (OMA1) is a metalloprotease located in the IMM, with OPA1 being one of its primary substrates (63). Under physiological conditions, OMA1 remains inactive but is rapidly activated during mitochondrial stress, such as MMP loss or excessive ROS production, negatively regulating OPA1 (64). It was demonstrated that leptin increased OPA1 expression by promoting the ubiquitin-mediated degradation of OMA1 via the GSK3 pathway, thereby enhancing the anti-apoptotic capacity of these cells (65).

### 2.3 Ubiquitination-mediated mitophagy

Similar to MK and MB, Mitophagy is another key process in the maintenance of line MH. Mitophagy is a cellular autophagic process that involves the selective sequestration and degradation of damaged or dysfunctional Mt, thereby maintaining the integrity of the mitochondrial network and cellular homeostasis (66, 67). Ubiquitination participates in mitophagy primarily through the PINK1/Parkin pathway, the PINK1/SYNPHILIN1/SIAH1 complex, as well as the interactions of Mitochondrial E3 Ub protein ligase 1 (MUL1) (68, 69).

#### 2.3.1 PINK1/Parkin pathway

The PINK1/Parkin pathway is the most widely studied mitophagy pathway and is a classic Ub-dependent pathway (70). PINK1, as a sensor of mitochondrial health, accumulates on the outer membrane of damaged Mt when they are compromised and activates Parkin, which in turn promotes the recruitment of Parkin to the Mt. PINK1 phosphorylates Ub on the Ser65 site of substrates on the mitochondrial outer membrane, a process that activates Parkin, allowing it to ubiquitinate numerous substrates on the mitochondrial outer membrane, thereby triggering selective autophagy (71). In healthy Mt, PINK1 is imported into the inner membrane, where its membrane-binding portion is cleaved by the protease presenilin-associated rhomboid-like (PARL) (72). The cleaved catalytic portion exposes unstable amino acid residues at the N-terminus and is rapidly degraded by the Ub-proteasome system (UPS) (72). This process is a crucial step in mitochondrial quality control, ensuring that only functionally intact Mt remains in the cell (73). When the function of PINK1 or Parkin is impaired, the removal of damaged Mt is hindered, leading to mitochondrial dysfunction and pathological changes in the organism (74). The loss of DJ-1 inhibits the recruitment of the selective autophagy receptor, synphilin, to depolarized Mt, further blocking PINK1/Parkin-mediated mitophagy (75). In addition to removing damaged mt, the PINK1/Parkin pathway also contributes to MB development. It has been shown that loss of PINK1/Parkin in nerve cells inhibits MB and ubiquitinates Parkin-interacting substrate, thereby relieving the inhibitory effect on PGC-1α (76).

#### 2.3.2 PINK1/SYNPHILIN1/SIAH1 complex

Similar to Parkin, seven in absentia homolog 1 (SIAH1) is also an E3 Ub ligase (77). Through forming a complex with PINK1 and synphiln1, SIAH1 promotes the recruitment of autophagic markers, Microtubule-associated protein one light chain 3 (LC3), and the lysosome-associated membrane protein 1 (Lamp1), thereby facilitating mitochondrial autophagy (78). LC3 is a key marker in the autophagy process. During autophagosome formation, the cytosolic form of LC3-I participates in a Ub-like reaction involving Autophagy-related (Atg) 7 and Atg3 (E1-like Ub-activating enzyme and E2-like Ub-conjugating enzyme), binding to phosphatidyl ethanolamine to form the lipidated form, LC3-II, which attaches to the autophagosomal membrane and serves as a structural protein of the autophagosome (79). LAMP1 is commonly used as a marker for lysosomes, and LAMP1-positive organelles are often referred to as lysosomal compartments. After the fusion of the autophagosome with the lysosome, lysosomal hydrolases can degrade the autophagosome’s contents, and LAMP1-labeled organelles participate in this degradation process (80).

#### 2.3.3 Involvement of MUL1

MUL1 participates in mitochondrial autophagy and mediates MM and MK (81). MUL1 mediates sodium selenite-induced mitochondrial autophagy and the stability of the autophagy-related protein Unc-51, Like autophagy activating kinase 1 (ULK1), during this process (82). Interacting with the ULK1/ATG13 complex, MUL1 promotes the formation of K48 polyubiquitin chains on ULK1, leading to its degradation via the UPS. In muscle cells, MUL1 mediates the degradation of MFN2 via the UPS while also inducing mitochondrial autophagy (82). Additionally, by promoting the Small Ub-like modifier conjugation (SUMOylation) of the highly dynamic protein Drp1, which is recruited to Mt, MUL1 enhances the stability of Drp1 on the mitochondrial surface, playing a critical role in the dynamic regulation of mitochondrial morphology (83). The regulatory effect of MUL1 on mitochondrial energy metabolism has also been observed. MUL1 regulates the protein levels of protein kinase Bβ and hypoxia-inducible factor 1-alpha (HIF-1α) through K48-specific polyubiquitination, and the loss of MUL1 leads to the accumulation and activation of these substrates, affecting mitochondrial respiration and resulting in a shift to a new metabolic and lipidomic state (84). This is evident in the fact that, compared to wild-type cells, MUL1(-/-) cells show impaired mitochondrial respiration and increased ATP production through glycolysis, indicating a metabolic shift from oxidative phosphorylation to glycolysis (84).

### 2.4 Ubiquitination in MM

Under physiological conditions, mitochondrial energy metabolism includes the tricarboxylic acid cycle, OXPHOS, and fatty acid oxidation (85). During these processes, ubiquitination plays a key role in regulating mitochondrial homeostasis through various metabolic products such as glucose, fatty acids, amino acids, and the electron transport chain (ETC) (86, 87).

#### 2.4.1 Glucose metabolism

Currently, the mechanisms by which ubiquitination participates in mitochondrial glucose metabolism under physiological conditions are not well understood. However, in certain pathological states, ubiquitination may play a role in reshaping mitochondrial glycolysis (88). In a study using a heart-specific promoter cTnT, the deletion of NEDD8-Activating enzyme E1 impaired cardiac oxidative metabolism and mitochondrial function, leading to the down-regulation of genes related to fatty acid utilization, while genes associated with glucose utilization were significantly up-regulated (89). Another study found that inhibition of polycomb repressive complex 1 reduced histone H2A ubiquitination (H2Aub) occupancy and, by suppressing ubiquitination, promoted the expression of Hsp27 (heat shock protein 27). Hsp27 enhances glycolysis during myocardial ischemia by activating the NF-κB/PFKFB3 signaling pathway, and it also reduces mitochondrial ROS production by interacting with Coenzyme Q9, inhibiting ferroptosis during reperfusion (90). Research progress shows that E3 ligases and deubiquitinating enzymes influence the Warburg effect in tumors by regulating glycolysis-related signaling pathways and transcription factors (91, 92). Phosphofructokinase platelet (PFKP) is a gene encoding a rate-limiting enzyme of glycolysis, and its role in mediating glycolytic regulation of tumor progression has been well-established in lung cancer and advanced prostate cancer (93, 94). HMG-CoA Reductase Degradation 1 (HRD1), as a metabolic enzyme, catalyzes the ubiquitination of PFKP and promotes its degradation, thereby inhibiting the expression and activity of PFKP in cancer cells and obstructing cell invasion and proliferation (95). The gut microbiota and its derivative metabolite taurocholic acid can epigenetically promote the glycolysis of Myeloid-derived suppressor cells by enhancing the monomethylation of the target gene H3K4 and inhibiting C-terminus of Hsc70-interacting protein-mediated PDL1 ubiquitination, which in turn suppresses the proliferation and function of effector T cells (96).

#### 2.4.2 Fatty acid metabolism

Under energy stress conditions, tumor cells mobilize lipids stored in lipid droplets and generate energy through mitochondrial fatty acid oxidation (β-oxidation) (97). The direct contact between lipid droplets promotes the hydrolysis of triglycerides in the lipid droplets into fatty acids and glycerol, which are then transported into theMt (98). In this process, nicotinamide adenine dinucleotide kinase (NADK) regulates fatty acid synthesis by maintaining the intracellular coenzyme nicotinamide adenine dinucleotide phosphate levels, thereby controlling lipid storage and metabolic homeostasis in lipid droplets (98). The knockdown of NADK affects the levels of acetyl-CoA, thereby regulating the acetylation modification of the key transcription factor PGC-1α and MB and influencing mitochondrial function and number by affecting CL synthesis (99). A Study found that mitochondrial Signal Transducer and Activator of Transcription 3 (STAT3) could reduce the ubiquitination and degradation of carnitine palmitoyltransferase 1a, thereby inhibiting fatty acid oxidation metabolism and reducing oxidative stress formation (100). In stem cells, the fatty acid synthesis regulated by the lipid synthesis enzyme Acetyl-CoA Carboxylase 1 can influence acetylation-mediated Fis1 Ub-proteasomal degradation by consuming Acetyl-Coenzyme A. At the same time, it can produce lipid products that drive the shift of Mt from a dynamic equilibrium to fission, thereby enhancing mitochondrial fission (101).

#### 2.4.3 Amino acid metabolism

Almost all amino acids are synthesized or degraded in the Mt (102). Impaired amino acid metabolism is associated with primary mitochondrial diseases and mitochondrial dysfunction disorders. For example, abnormalities in branched-chain amino acid metabolism are closely related to the progression of diseases such as diabetes (103), atherosclerosis (104), and cancer (105). Wang T et al. (106) conducted experiments on amino acid starvation in tumor cells, assessing the levels of K48-polyubiquitinated proteins in cultured cells after starvation. They found that short-term starvation promoted protein ubiquitination, but after prolonged treatment, there was a significant decrease. This suggests that early amino acid depletion promotes protein ubiquitination, while later stages lead to the degradation of these polyubiquitinated proteins. This phenomenon may be related to the energy depletion induced by amino acid starvation, which in turn triggers mitochondrial autophagy. Current research generally considers the UPS and autophagy-lysosome systems to have distinct functions, but the two systems can interact and influence each other. Amino acid starvation may simultaneously affect the UPS (107), protease activity in mTOR-inhibited Human Embryonic Kidney 293 cells, and polyubiquitination of the 26S proteasome (108). These pathways can all lead to autophagy to varying extents, with amino acid starvation being an important ubiquitination-mediated mechanism for regulating mitochondrial homeostasis (109).

The mechanistic target of rapamycin complex 1 (mTORC1) is a serine/threonine kinase that integrates various environmental signals to regulate cell growth and metabolism. Activation of mTORC1 requires binding to the lysosome through the Ragulator-Rag complex. One essential component of Ragulator, mTOR Activator 1 (LAMTOR1), undergoes dynamic ubiquitination modifications in response to the abundance of amino acids. The E3 ligase TRAF4 directly interacts with Late Endosomal/Lysosomal Adaptor and MAPK and LAMTOR1 and catalyzes polyubiquitination at the K151 site with K63 linkages. This ubiquitination promotes the binding of LAMTOR1 to Rag GTPases and enhances the activation of mTORC1 (110).

#### 2.4.4 ETC

ETC in Mt consists of a series of protein complexes (NADH dehydrogenase, succinate dehydrogenase, cytochrome c reductase, and cytochrome c oxidase) located on the IMM. These complexes are responsible for transferring electrons from one complex to another, ultimately transferring electrons to oxygen, resulting in the formation of water (111). Ubiquitination plays a role in the ETC mechanism, primarily through the targeting of specific proteins. The epigenetic regulator Unfolded Protein Response Factor 1 modulates K27 ubiquitination through NLRP14, thereby maintaining the stability of the mitochondrial Na+/Ca2+ exchanger protein Mitochondrial Sodium/Calcium/Lithium Exchanger, which ensures the stability of mitochondrial morphology and function (112). The contact sites between the endoplasmic reticulum membrane and the mitochondrial membrane, known as mut-associated membranes, represent multifunctional microdomains involved in mitochondrial homeostasis (113). Mt-associated membrane-specific E3 Ub ligases can ubiquitinate nascent proteins, thereby activating TANK binding kinase 1 on the Mt-associated membrane (114), leading to the degradation of ribosomal proteins and disrupting the overall mitochondrial homeostasis. Meanwhile, the Mt-associated membrane is closely linked to Ca2+ homeostasis. Upon ubiquitination of inositol 1,4,5-trisphosphate receptor type 2, the mitochondrial outer membrane protein Fundc1 loses its binding site, promoting increased mitochondrial Ca2+, mitochondrial fragmentation, and apoptosis (112).

In addition, RNA molecules also have a significant association with ETC. BDNF-AS is a natural antisense long non-coding RNA of brain-derived neurotrophic factor (BDNF) (115). The expression of BDNF-AS is significantly positively correlated with Voltage dependent anion channel 3 (VDAC3) expression. Previous studies have shown that VDAC3 influences cellular ferroptosis by regulating mitochondrial iron ion flux (116), but the mechanism by which BDNF-AS regulates VDAC3 expression is still unclear. Circular RNA Microtubule crosslinking factor 1 can promote its development by inhibiting the Ub-mediated degradation of the mitochondrial protein complement C1q binding protein and mediating β-catenin activation (117). Under various stress-induced cellular senescence conditions, the expression of SIRT1 protein is down-regulated through Ub-mediated proteasomal degradation (118), a process typically involving Ub-dependent proteasomal degradation. However, some studies suggest that DNA damage-induced cellular senescence follows the autophagosome-lysosome pathway, which may be linked to mitochondrial homeostasis imbalance caused by DNA damage, leading to a decline in the NAD+-dependent biological function of SIRT1, affecting the ubiquitination binding process (119).

**Figure 2:**
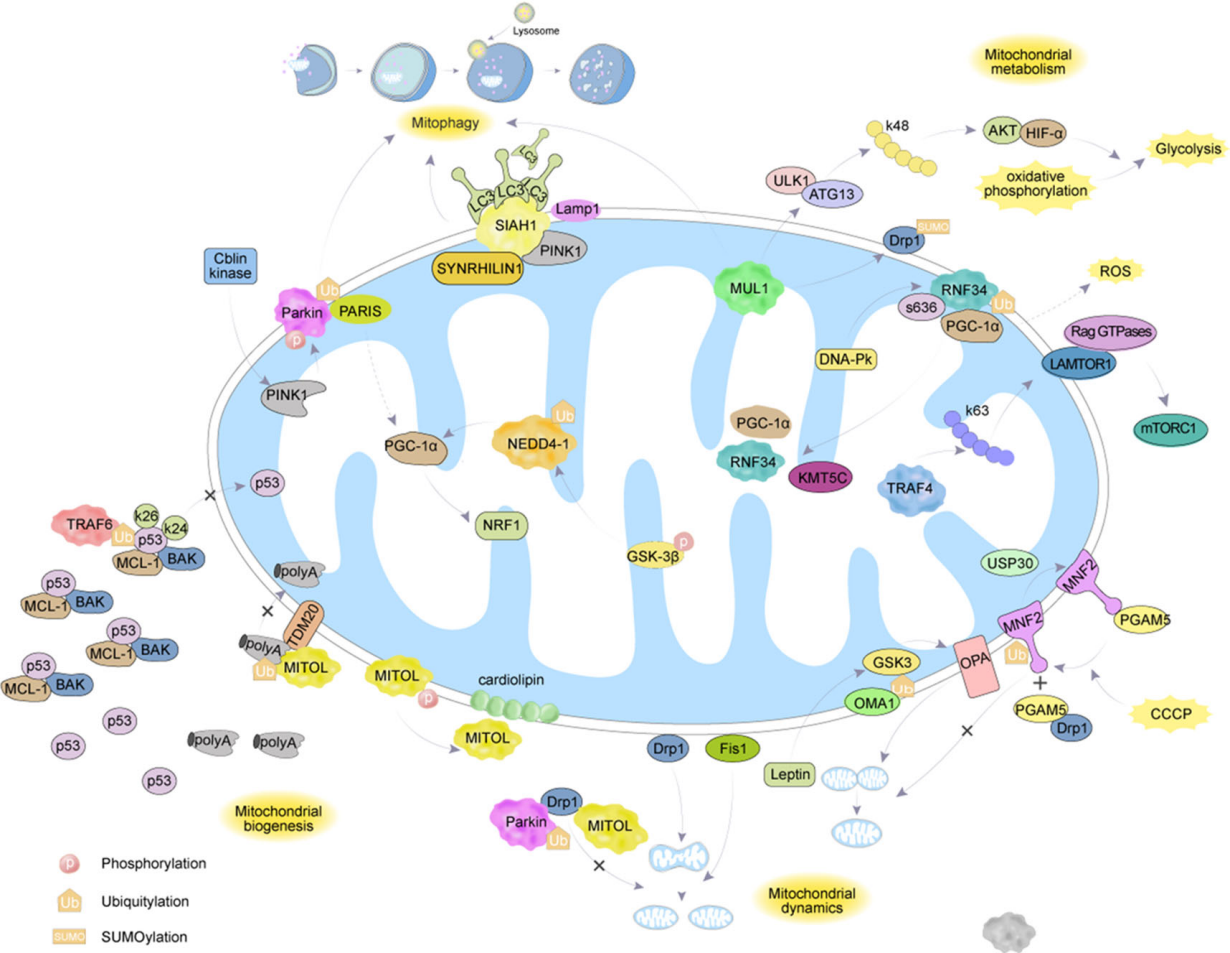
The regulatory mechanisms of ubiquitination enzymes and deubiquitination enzymes on MH.

The mechanism of interaction between ubiquitination and deubiquitination targeting MH is shown in **Figure 2** and **Table 1**.

**Table 1:** Ubiquitinating enzymes targeting MH.

Ub/DUB	Full name	Type
SIAH1	Siah E3 Ubiquitin Protein Ligase 1	E3 ubiquitin ligase
Parkin	Parkin rbr E3 ub protein Ligase	E3 ubiquitin ligase
MUL1	Mitochondrial Ubiquitin Ligase Activity Factor 1	E3 ubiquitin ligase
RNF34	Ring Finger Protein 34	E3 ubiquitin ligase
NEDD4-1	Neural Precursor Cell Expressed, Developmentally Down-regulated Protein 4-1	E3 ubiquitin ligase
MITOL/MARCH5	Mitochondrial ubiquitin ligase/Membrane-Associated RING-CH-type finger protein	E3 ubiquitin ligase
TRAF6	TNF receptor-associated factor 6	E3 ubiquitin ligase
TRAF4	TNF receptor-associated factor 4	E3 ubiquitin ligase
USP30	Ubiquitin-specific Protease 30	DUBs

## 3 Ubiquitination targeting MH in the progression of GI tumors

### 3.1 HCC

In HCC, In HCC, ubiquitination targeting the MH mechanism with several therapeutic agents was found (**Table 2**). PINK1/Parkin-mediated mitophagy plays a crucial role. Cyclin-dependent kinase 9 (CDK9) activates SIRT1 and promotes the stabilization of PINK1 protein through its mediated deacetylation, but this effect is blocked by Wogonin (122). Matrine, a traditional Chinese medicine (TCM), triggers mitochondrial dysfunction and induces the upregulation of PINK1/Parkin through phosphatase and PTEN. The elevation of the p62 and LC3-II/I ratio suggests that Matrine acts as both an inducer of autophagy and an inhibitor of autophagosome-lysosome formation, while the blockade of autophagy promotes Matrine-induced cell death (124). The TCM Quercetin (123) upregulates the expression of PINK1/Parkin in Huh7 and Hep3B cells, thereby exerting its anti-cancer effects in HCC. The hepatitis B virus (HBV)-encoded X protein (HBx) plays a key role in inducing HCC (129). Studies have found that thyroid hormone (TH) induces the ubiquitination of mitochondrial-related HBx through PINK1/Parkin and triggers selective mitophagy, thereby inhibiting HBx-promoted ROS and carcinogenesis (125). Lipid metabolism disorder is one of the important characteristics of HCC. Ubiquitin conjugating enzyme E2 O (UBE2O), as an E2 enzyme, has been found to promote HCC progression with high expression. Meanwhile, UBE2O interacts with the mitochondrial β-oxidation enzyme and mediates its ubiquitination and degradation, thereby regulating lipid metabolism reprogramming under the action of E2 and E3 enzyme activities (126). Studies have shown that the VDAC1 inhibitor Novobiocin can reduce the mono-ubiquitination level of VDAC1 K274, and subsequent mutation of this site weakens the interaction between Hsp90α-VDAC1, increases the oligomerization of VDAC1, and thus affects the progression of HCC (120). Crosstalk between some forms of cell death has also been found in HCC. During ferroptosis, 3-hydroxy-3-methylglutaryl-CoA reductase (HMGCR) is primarily localized to the Mt, but after treatment with pyroptosis inducers, HMGCR translocates to the endoplasmic reticulum. BRCA1/BRCA2-containing complex subunit 36 deubiquitinates HMGCR through DUB activity and inhibits ferroptosis while promoting pyroptosis (127). Transarterial chemoembolization (TACE) is the main treatment method for advanced liver cancer, but postoperative hypoxia can easily worsen the patient’s condition. The hypoxic environment caused by TACE leads to overexpression of S100 calcium-binding protein A9, which forms trimers as a penta-glycine motif protein, triggers mitochondrial fission and ROS production through the deubiquitination and stabilization of PGAM5, and ultimately promotes the growth and metastasis of HCC (121).

**Table 2:** Ubiquitination targeting the MH mechanism in HCC.

MH	Medicine	Targets	Ubiquitination	Deubiquitination	Main findings	References
MB	/	Hsp90	VDAC1(K274)	/	Hsp90 promotes cell apoptosis associated with VDAC1 oligomerization by reducing VDAC1 protein K274 monubiquitination	(120)
MK	/	S100A9	/	PGAM5	As a scaffold, S100A9 recruits ubiquitin-specific peptidase 10 and phosphoglycerate mutase family member 5 (PGAM5) to form a trimer, causing deubiquitination and stabilization of PGAM5, leading to mitochondrial fission and reactive oxygen species production, thereby promoting HCC growth and metastasis	(121)
Mitophagy	/	CDK9 targets SIRT1-FOXO3-BNIP3	PINK1-Parkin	/	CDK9 inhibition blocks the initiation of PINK1-PRK1-mediated mitophagy by regulating the SIRT1-FOXO3-BNIP3 axis and enhances the therapeutic efficacy of treatments involving mitochondrial dysfunction in HCC	(122)
	Quercetin	SIRT1	PINK1-Parkin	/	Quercetin up-regulates the expression of PINK1 and PARK2, which are the regulators of mitophagy, and enhances the colocalization of mitochondria and lysosomes to promote autophagy	(123)
	Sanguinarine	/	PTEN-PINK1-Parkin	/	Sanguinarine promoted mitochondrial apoptosis by blocking mitophagy through PINK1-Parkin	(124)
	TH	HBx	PTEN-PINK1-Parkin	/	TH simultaneously induces mitochondrial biogenesis and HBx-targeted mitochondrial autophagy, thereby inhibiting HBx-promoted ROS and carcinogenesis	(125)
MM	/	HADHA	UBE2O	/	UBE2O promotes lipid metabolism reprogramming and liver cancer progression by mediating HADHA ubiquitination	(126)
	/	BRCC36	/	HMGCR	BRCC36 deubiquitinates HMGCR through the activity of deubiquitinating enzymes, as well as inhibiting ferroptosis and promoting pyroptosis. In addition, BRCC36 acts as an oncogene in HCC, promoting cancer cell proliferation, migration, invasion, and tumor growth	(127)
	Ponicidin	Keap1-PGAM5	PGAM5	/	ponicidin targets Keap1 and promotes the formation of the Keap1-PGAM5 complex, ubiquitinizes PGAM5, and activates the cysteine-dependent mitochondrial pathway, leading to mitochondrial damage and ROS production	(128)

### 3.2 PAAD

In PAAD, the enhancement of ferroptosis or drug sensitivity through ubiquitination has garnered attention (**Table 3**). Due to the dense stroma of PAAD tumors, most tumor-targeting drugs are not sensitive to PAAD treatment (136). As a first-line oncology drug, gemcitabine exhibits a significant chemoresistance phenomenon in pancreatic cancer (137). Stomatin like 2 (STOML2), also known as SLP-2, is a protein located in the mitochondrial inner membrane that participates in maintaining mitochondrial stability (138). Studies have found (113) that increased expression levels of STOML2 can stabilize PARL, thereby preventing PINK1-dependent mitophagy induced by gemcitabine (131). In mammals, ULK1 is a key component of the autophagy initiation complex (139). In PAAD, the E3 ligase NEDD4 like E3 ubiquitin protein ligase NEDD4L binds to ULK1 and is involved in its ubiquitination regulation. In NEDD4L knockout cells, genetic or pharmacological inhibition of ULK1 or Solute Carrier Family 1 Member 5 (SLC1A5/ASCT2) can sensitize PAAD cells, especially under nutrient deprivation conditions (135). Ferroptosis activator imidazole erastin (IKE) upregulates its E3 Ub ligase RANBP2-type and C3HC4-type zinc finger containing 1 (RBCK1), and the knockdown of RBCK1 enhances the cytotoxic effect of IKE on PAAD cells (134). This is attributed to the interaction between RBCK1 and MFN2, leading to polyubiquitination and promoting proteasomal degradation under ferroptotic stress, which results in reduced ROS and lipid peroxidation. Another study indicates that the PINK1-PARK2 pathway-mediated degradation of SLC25A37 and SLC25A28 increases mitochondrial iron accumulation, leading to HIF-1α dependent Warburg effect and AIM2 inflammasome activation in tumor cells, promoting the release of high mobility group box 1 and further inducing the expression of CD274/PD-L1 (133). Galactan RN0D, isolated from the TCM Sanqi, has been identified as an activator of the PINK1/Parkin pathway, ultimately activating cytotoxicity in tumor cells (132). Ubiquitination-mediated Mt gene transcription has also been observed in PAAD. As a core component of endoplasmic reticulum-associated protein degradation, the HRD1-SEL1L complex, when increased, reduces the stability of the mitochondrial protein AlkB homolog 1, leading to impaired transcription of mitochondrial DNA-encoded genes (140). The SIRT4 agonist entinostat reverses this process (130), possibly by deacetylating lysine 547 of SEL1L and increasing the protein levels of HRD1.

**Table 3:** Ubiquitination targeting the MH mechanism in PAAD.

MH	Medicine	Targets	Ubiquitination	Deubiquitination	Main findings	References
MB	Entinostat	SIRT4	SEL1L-HRD1-ALKBH1	/	SIRT4 deacetylates lysine 547 of SEL1L and increases protein levels of the E3 ubiquitin ligase HRD1. Increased SEL1L-HRD1 complex decreases the stability of the mitochondrial protein ALKBH1. Upon down-regulation of ALKBH1, transcription of mitochondrial DNA-encoding genes is blocked, leading to mitochondrial damage.	(130)
Mitophagy	Gemcitabine	STOML2	PARL/PINK1	/	STOML2 regulates autophagy through the PARL/PINK1 pathway, thereby reducing the chemoresistance of pancreatic cancer. Overexpression of STOML2 as a targeted therapy may help sensitize gemcitabine in the future.	(131)
	RN0D	/	PTEN-PINK1-Parkin	/	RN0D is identified as an activator of the PTEN-induced kinase 1 (PINK1)/Parkin pathway, ultimately activating cytotoxic mitophagy in tumor cells.	(132)
MM	/	SLC25A37/SLC25A28	PINK1-PARK2	/	The PINK1-PARK2 pathway mediates the degradation of SLC25A37 and SLC25A28, increasing mitochondrial iron accumulation, leading to HIF1A-dependent Warburg effect and AIM2-dependent inflammasome activation in tumor cells. AIM2-mediated HMGB1 release further induces the expression of CD274/PD-L1. Therefore, in PINK1-/- and PARK2-/- mice, pharmacological administration of mitochondrial iron chelators, anti-HMGB1 antibodies, or genetic knockout of Hif1a or Aim2 can protect against the development of pancreatic tumors. Low expression of PARK2 and high expression of SLC25A37 and AIM2 are associated with poor prognosis in patients with pancreatic cancer.	(133)
	Erastin	MFN2	RBCK1	/	The ferroptosis activator erastin (IKE) induces the upregulation of E3 ubiquitin ligase RBCK1 expression in PDAC cells at the transcriptional or translational level. *In vitro*, knockdown or absence of RBCK1 makes PDAC cells more susceptible to IKE-induced ferroptosis. In a mouse xenograft model, RBCK1 gene knockout increases the killing effect of ferroptosis inducers on PDAC cells. Mechanistically, RBCK1 interacts with and polyubiquitinates the key regulator of mitochondrial dynamics, mitofusin 2 (MFN2), to promote its proteasomal degradation under ferroptosis stress, leading to reduced mitochondrial ROS generation and lipid peroxidation.	(134)
	/	ULK1	NEDD4L	/	NEDD4L can ubiquitinate and degrade ULK1. After knockdown of NEDD4L, the autophagy activity in cells is enhanced, and the cellular oxygen consumption rate and mitochondrial membrane potential increase, maintaining the fusion state of mitochondria to cope with metabolic stress.	(135)

### 3.3 CRC

In CRC, several ubiquitination mechanisms have also been identified (**Table 4**). The p53 is one of the most important tumor suppressors, which inhibits the formation and development of tumors by regulating the expression of various genes, including those that promote cell cycle arrest and apoptosis (151). In CRC, p53 ubiquitination plays a significant role. A protein named UBX domain-containing protein 2A promotes the carboxy-terminal ubiquitination of Mortalin-2 in an Hsp70 interaction-dependent manner, reducing Mortalin-2 levels, which not only inactivates p53 but also directly promotes tumor cell invasion and migration (144). The mitochondrial antiviral signaling (MAVS) protein promotes p53-dependent cell death in response to DNA damage (143). MAVS is underexpressed in CRC and inhibits p53 ubiquitination by blocking the formation of the p53-MDM2 complex (143). Lipoic acid (LA) is a dithiol compound with redox activity and is an essential cofactor for mitochondrial oxidative decarboxylation (152). The p53 is found to be ubiquitinated and degraded by the proteasome mechanism after LA treatment, a process that does not involve the MDM2. Interestingly, the combined application of LA and anticancer drugs (doxorubicin, 5-fluorouracil) attenuates the stabilization of p53-mediated p21 and exerts a synergistic cytotoxic effect on CRC cells in a p53-dependent manner (153). Dihydroartemisinin downregulates the expression of the mitochondrial inner membrane scaffold protein anti-proliferative protein 2 in a Ub-dependent manner and blocks the downregulation of p53 and p21, thereby enhancing the cytotoxicity of oxaliplatin in CRC (154). TRAF6 promotes the K63-linked ubiquitination of p53 at K24 in the cytoplasm to limit the interaction between p53 and MCL-1/BAK, thereby restricting the mitochondrial translocation of p53 and spontaneous apoptosis. Additionally, TRAF6 promotes the K63-linked ubiquitination and transactivation of nuclear p53 by recruiting p300 to acetylate p53 (35). The PINK1/Parkin pathway has also been identified in CRC. SIRT3 is highly expressed in CRC with mitochondrial dysfunction, leading to PINK1/Parkin-mediated mitophagy. Targeting histone H2Aub ubiquitination at K119 reduces it, thereby enhancing DNA damage repair induced by radiation (147). Delta-valentine, as an emerging dietary metabolite, targets SIRT3 to participate in the process (146), while Aloe Gel Polysaccharides mediate the PINK1/parkin pathway in a ROS-dependent manner (148). Non-coding RNA targeting ubiquitination to regulate mitochondrial homeostasis mechanisms in CRC has been discovered for the first time. Non-coding RNA piR-823 interacts with PINK1, promoting its ubiquitination and proteasome-dependent degradation, thereby alleviating mitophagy, a mechanism reversed by Ant-823, which promotes Parkin activation (145). Regulation of the ETC and MM has also been identified in CRC. Receptor-interacting protein kinase 1 (RIPK1) interacts with the mitochondrial calcium uniporter (MCU), promoting cell proliferation by increasing mitochondrial calcium uptake and energy metabolism. The ubiquitination site of RIPK1 (RIPK1-K377) is a key site for interaction with MCU and the promotion of cell proliferation (150). As a molecular chaperone of Hsp90, Tumor necrosis factor receptor-associated protein 1 regulates the glycolytic enzyme phosphofructokinase-1 (PFK1) to maximize lactate production, balancing low OXPHOS. This depends on the interaction between TRAP1 and PFK1, which favors the glycolytic activity of PFK1 and prevents its ubiquitination/degradation (155). The interaction between membrane glycoprotein CD36 and glypican 4 (GPC4) induces proteasome-dependent ubiquitination and degradation of GPC4 in CRC cells, reducing the high addiction of CRC cells to glucose or glycolytic inhibition through mitochondrial reduction, i.e., reducing the expression of glycolytic target genes GLUT1, HK2, PKM2, and LDHA, thereby inhibiting the energy metabolism and growth of tumor cells (149). Deubiquitination is the reverse process of ubiquitination (142). USP36 exerts its pro-apoptotic function by targeting cIAP1 and survivin, and it has been found that USP36 can be degraded through polyubiquitination, although the E3 ligase responsible for this process remains unidentified (156).

**Table 4:** Ubiquitination targeting the MH mechanism in CRC and IBD.

Cancer	MH	Medicine	Targets	Ubiquitination	Deubiquitination	Main findings	References
CRC	MB	Compound 8	PKM2	VDAC3	/	Succination-mediated translocation of PKM2 to mitochondria under glucose starvation conditions plays a role in the cell’s switch from a proliferative mode to a survival mode, and vice versa. Mitochondrial PKM2 inhibits ubiquitination-mediated degradation of VDAC3 and increases mitochondrial permeability, producing more ATP to maintain cell survival under nutrient depletion. In human colorectal cancer, the upregulation of mitochondrial PKM2 is positively correlated with the upregulation of VDAC3.	(141)
		/	TRAF6	p53	/	TRAF6 promotes the ubiquitination of p53 in the cytoplasm, thereby limiting the mitochondrial translocation of p53 and spontaneous apoptosis.	(35)
		/	survivin and cIAP1	/	USP36	USP36 binds to and removes the lysine-11 linked ubiquitin chains of cIAP1 and the lysine-48 linked ubiquitin chains of survivin, thereby preventing protein degradation. Overexpression of USP36 can disrupt the formation of the XIAP-second mitochondrial-derived caspase activating factor (SMAC) complex and promote the ubiquitination of receptor-interacting protein kinase 1 (RIPK1), confirming that USP36 can inhibit both intrinsic and extrinsic apoptosis by deubiquitinating survivin and cIAP1.	(142)
		/	MDM2	p53	/	The MAVS protein can inhibit the ubiquitination of p53, promoting p53-dependent cellular damage.	(143)
		veratridine	mot-2-CHIP	UBXN2A	/	UBXN2A promotes the carboxy-terminal ubiquitination of mot-2 that is dependent on the HSP70-interacting protein (CHIP). UBXN2A increases the proteasomal degradation of mot-2. Subcellular compartmentalization experiments show that UBXN2A can reduce the expression levels of mot-2 and its chaperone HSP60. The existence of a multi-protein complex of UBXN2A, CHIP, and mot-2 indicates that UBXN2A and CHIP have synergistic tumor suppressor activity in tumors enriched with mot-2.	(144)
	Mitophagy	/	piR-823	PINK1	/	piR-823 inhibits PINK1-mediated mitophagy.	(145)
		δVB	/	PINK1-Parkin		δVB activates mitochondrial apoptosis through the PINK1/Parkin pathway, with observed upregulation of PINK1, Parkin, and LC3B protein levels.	(146)
		/	SIRT3	PINK1-Parkin	/	SIRT3 is highly expressed in colorectal cancer cells with mitochondrial dysfunction, leading to PINK1/parkin-mediated mitophagy.	(147)
		AGP	/	PINK1-Parkin	/	AGP induces cytotoxic mitophagy in CT26 cells through the ROS-associated PINK1/Parkin pathway and TFEB activation.	(148)
	MM	/	CD36	GPC4	/	cd36 -GPC4相The CD36-GPC4 interaction promotes proteasome-dependent ubiquitination of GPC4, thereby inhibiting the β-catenin/c-myc signaling pathway and downstream glycolytic target genes GLUT1, HK2, PKM2, and LDHA. Furthermore, the knockout of CD36 significantly increased the occurrence of colorectal tumors in inflammation-induced CRC models and ApcMin/+ mouse models.	(149)
		/	RIPK1-MCU	RIPK1(k377)	/	RIPK1-K377 is a key site for interaction with MCU, and upon binding, it promotes cell proliferation by increasing mitochondrial Ca2+ uptake and energy metabolism.	(150)

IBD is a chronic relapsing inflammatory disorder associated with an increased risk of developing CRC (157). Compared with sporadic CRC, IBD-related CRC typically occurs at a younger age, progresses more rapidly, and has a worse prognosis (158). Therefore, focusing on the pathogenesis of IBD can help prevent the development of CRC in advance. Studies have shown that mitochondrial dysfunction activates pyroptosis through multiple pathways, thereby exacerbating the occurrence of IBD (159). Mitochondrial dysfunction, combined with impaired autophagy, leads to increased ROS levels, which activate the Nucleotide-binding oligomerization domain, Leucine-rich repeat, and Pyrin domain-containing protein 3 (NLRP3) inflammasome and subsequently induce pyroptosis (160). Meanwhile, defects in mitochondrial respiration lead to cellular energy metabolism disorders, making cells more prone to pyroptosis (160). Pyroptosis of intestinal epithelial cells disrupts the intestinal barrier, rendering the gut more susceptible to attacks from pathogens and inflammatory factors. Additionally, the pore-forming action of Gasdermin proteins, which causes cell membrane rupture and the release of large amounts of inflammatory factors (such as IL-1β and IL-18), further intensifies the intestinal inflammatory state (159). Research has found that the E3 Ub ligase gp78 mediates mixed ubiquitination of NLRP3, inhibiting its activity by preventing the oligomerization and subcellular translocation of the nucleotide-binding and oligomerization domain of NLRP3, thereby reducing inflammasome activation and its detrimental effects (161). Parkin-driven mitophagy and inhibition of NLRP3 inflammasome activation in the colon exert a protective effect against DSS-induced colitis in mice (162). Moreover, in IBD, the epigenetic modifier SET and MYND Domain containing protein 5 (SMYD5) regulates toll-like receptor 4 target genes in macrophages at the K20 site (163). This regulation increases the risk of IBD progression to CRC by mediating PGC-1α ubiquitination and degradation through methylation, thereby inhibiting MB.

### 3.4 GC

For GC patients, clinical drug resistance has always been a limitation in late-stage treatment. X-ray repair cross-complementing 1 (XRCC1) is a key regulator of cisplatin-induced DNA damage and apoptosis (164). Thioredoxin-like 1 mediates cisplatin resistance by negatively regulating the expression of XRCC1 through the UPS (165). Myocyte enhancer factor 2A activates PGC1α transcription and inhibits Kelch-like ECH-associated protein 1, reducing the ubiquitination and degradation of NRF2, thereby regulating ROS levels and mediating GC cisplatin resistance (166). Ferroptosis resistance is one of the key factors leading to GC drug resistance. Studies have shown that SRY-box transcription factor 13 (SOX13) promotes the protein reshaping of ETC complexes by directly transactivating Supercomplex assembly factor 1, leading to the assembly of supercomplexes, mitochondrial respiration, mitochondrial energetics, and increased chemo- and immuno-resistance (167). Zanamivir restores the ferroptosis resistance phenotype by directly targeting SOX13 and promoting the ubiquitination and degradation of SOX13 mediated by a tripartite motif containing 25 (TRIM25) (167). Research has found that LncRNA BDNF-AS can affect the ubiquitination modification of VDAC3 by FBXW7 by recruiting WD repeat-containing protein 5 (168). The USP7 can stabilize Heterogeneous nuclear ribonucleoprotein A1 in cancer-associated fibroblasts through deubiquitination, leading to increased secretion of exosomal miR-522, thereby inhibiting ferroptosis and promoting acquired drug resistance in GC (169), primarily by targeting arachidonate 15-lipoxygenase and blocking the accumulation of lipid ROS in Mt. In TCM treatment, the compound herbal medicine Huachansu induces apoptosis in GC cells by increasing ROS levels and inhibiting USP activity (170). Mechanisms and targets are shown in **Table 5**.

**Table 5:** Ubiquitination targeting the MH mechanism in GC.

MH	Medicine	Targets	Ubiquitination	Deubiquitination	Main findings	References
MB	/	MEF2A	KEAP1-NRF2	/	MEF2A activates the transcription of PGC1, increasing mitochondrial biogenesis. MEF2A inhibits the transcription of KEAP1, reducing the ubiquitination and degradation of NRF2, and activating the KEAP1/NRF2 signaling pathway, thereby regulating reactive oxygen species levels and maintaining the homeostasis of the mitochondrial biogenesis process.	(166)
	Cisplatin	TXNL1	XRCC1	/	XRCC1 is a key regulator of cisplatin-induced DNA damage and apoptosis. TXNL1, a member of the thioredoxin family, negatively regulates the expression of XRCC1 through the ubiquitin-proteasome pathway.	(165)
MM	/	BDNF-AS-WDR5-FBXW7	VDAC3	/	BDNF-AS regulates the expression of FBXW7 by recruiting WDR5, thereby affecting the transcription of FBXW7; FBXW7 ubiquitinates and regulates the protein expression of VDAC3.	(168)
	Zanamivir	SOX13	TRIM25	/	Zanamivir targets SOX13 and promotes the ubiquitination and degradation of SOX13 mediated by TRIM25, inhibiting the assembly of the mitochondrial respiratory chain supercomplex and restoring ferroptosis sensitivity.	(167)
	Cisplatin and paclitaxel	mir-522-ALOX15	/	hnRNPA1(USP7)	hnRNPA1 is found to mediate the packaging of miR-522 into exosomes, and USP7 stabilizes hnRNPA1 by deubiquitination. Cisplatin and paclitaxel promote the secretion of miR-522 from cancer-associated fibroblasts (CAFs) by activating the USP7/hnRNPA1 axis, leading to the suppression of ALOX15 and a reduction in lipid-ROS accumulation in cancer cells, ultimately leading to decreased chemotherapy sensitivity.	(169)
	CCMH	PI3K/Akt and MAPK	UPS	/	CCMH affects the ROS pathway, ubiquitin-proteasome system, PI3K/Akt, and MAPK signaling pathways. CCMH significantly increases the level of ROS in gastric cancer cells, and NAC can reverse the effect of CCMH on ROS levels in gastric cancer cells. NAC antagonizes the apoptotic induction of CCMH. CCMH can significantly reduce the activity of the 20S proteasome in gastric cancer cells. CCMH also regulates the expression of key proteins in the PI3K/Akt and MAPK signaling pathways.	(170)

### 3.5 ESCA

In ESCA, OTU deubiquitinase 1 is a deubiquitinating enzyme that regulates the apoptosis-inducing factor (AIF), capable of ubiquitinating AIF at K244, impairing mitochondrial oxidative phosphorylation, and reducing cell viability. Additionally, its deubiquitination at K255 enhances AIF’s binding capacity to DNA, promoting the occurrence of parthanatos (171). On the other hand, the E3 Ub ligase Itch plays a significant role in TNF-related apoptosis-inducing ligand-mediated apoptosis in ESCA. Knockdown of Itch leads to resistance to TNF-related apoptosis-inducing ligand-mediated apoptosis and significantly alters mitochondrial morphology, increasing mitochondrial cholesterol content. High cholesterol levels reduce membrane fluidity, further intervening in mitochondrial dynamic homeostasis (172). Apart from cholesterol, proteins are also an important factor affecting mitochondrial dynamic homeostasis. Syntaphilin (SNPH) is a static mitochondrial anchor protein primarily expressed in the brain, playing a crucial role in neurotransmitter release and MK. In various tumor cells, SNPH is downregulated or even silenced, leading to the redistribution of Mt from the perinuclear area to the cell periphery, resulting in increased tumor cell migration and invasion (173). Studies have found that CUL1 can ubiquitinate SNPH, disrupting mitochondrial dynamic homeostasis and promoting tumor metastasis and radioresistance (174). In the transformation of ESCA keratinocytes, cells with high CD44 expression exhibit a series of mitochondrial autophagy characteristics: mitochondrial fragmentation, reduced mitochondrial content, and Parkin mitochondrial translocation (175). Mechanisms and targets are shown in **Table 6**.

**Table 6:** Ubiquitination targeting the MH mechanism in ESCA.

MH	Medicine	Targets	Ubiquitination	Deubiquitination	Main findings	References
MK	/	CREB-SNPH	UPS	/	Ubiquitin-proteasome degradation and histone modification promote the downregulation of SNPH in RR ESCC cells. Dephosphorylation of CREB promotes the re-expression of SNPH, which induces radiosensitization. Moreover, the expression of SNPH is related to the radiotherapy efficacy in esophageal squamous cell carcinoma and is an independent prognostic factor for patients with esophageal squamous cell carcinoma.	(174)
Mitophagy	/	CD44	Parkin	/	Cells with high CD44 expression exhibit a series of mitochondrial autophagy characteristics: mitochondrial fragmentation, reduced mitochondrial content, and Parkin translocation to mitochondria.	(175)
MM	/	OTUD1	/	AIF(K244)	OTUD1 can deubiquitinate AIF at position K244, disrupt mitochondrial structure, and impair OXPHOS, promoting the function of AIF in mitochondrial respiration, and inducing a shift in cellular metabolism towards glycolysis.	(171)
	/	COP1/ZRANB1	MITF	/	COP1 and ZRANB1 jointly regulate the ubiquitination status of MITF, maintaining the stability of mitochondrial structure and function.	(176)
	/	Itch	STARD1	/	After Itch is knocked out, the morphology of mitochondria changes significantly, and cholesterol content increases. Itch may stabilize STARD1, increase the input of cholesterol to mitochondria, thereby inhibiting Bax activation and the release of cytochrome c.	(172)

## 3 Discussion

In summary, we have summarized the various aspects of MH regulation by different ubiquitinases and deubiquitinases in current scientific research, including MB, mitophagy, and the MM involved in these processes. In gastrointestinal tumors, through the ubiquitination regulation of MH, we have discovered different cell death crosstalk mechanisms, such as mitophagy, ferroptosis, and apoptosis, tumor drug resistance mechanisms, metabolic reprogramming, and new targets for TCM treatment. However, the specific roles of these mechanisms and the potential crosstalk between signaling pathways in different tumor types have not yet been fully elucidated. Clinical treatment results suggest that single-target therapies may not be sufficient for gastrointestinal tumors, and the development of new drugs is needed. Proton beam therapy (PBT) has been shown to inhibit colon cancer metastasis by stimulating mitochondrial biogenesis through the upregulation of PGC-1α and its co-transcription factors (NRF1α/ERRα). Additionally, compounds like hydroxytyrosol (HTyr) can promote mitochondrial biogenesis by increasing PGC-1α expression, offering potential as adjuvant anticancer agents. Advances in gene-editing technologies, such as CRISPR/Cas9, offer the potential to directly target mitochondrial dysfunction by correcting genetic defects or modulating key regulatory pathways. Although clinical translation is still in its infancy, preclinical studies have demonstrated the feasibility of these approaches. In addition, current research has not determined the mechanisms by which ubiquitination regulation of MH is involved in the immune evasion of gastrointestinal tumors, which may require further experimental data support.

## Glossary

MH: Mitochondrial Homeostasis
MB: Mitochondrial Biogenesis
MM: Mitochondrial Metabolism
MK: Mitochondrial Kinetics
GI: Gastrointestinal
Mt: Mitochondria
mtDNA: Mitochondrial DNA
HCC: Hepatocellular Carcinoma
ESCA: Esophageal Cancer
PAAD: Pancreatic Cancer
GC: Gastric Cancer
CRC: Colorectal Cancer
ROS: Reactive Oxygen Species
BCL-2: B-cell lymphoma/leukemia-2
IBD: Inflammatory Bowel Disease
Ub: Ubiquitin
DUBs: Deubiquitinating enzymes
USP: Ub-specific protease
UPS: Ubiquitin-Proteasome System
PGC-1α: peroxisome proliferator-activated receptor gamma coactivator-1 alpha
OXPHOS: Oxidative Phosphorylation
NRFs: Nuclear Respiratory Factors
MITOL1/MARCH5: Mitochondrial ubiquitin ligase/Membrane-Associated RING-CH-Type Finger 5
PolγA: DNA Polymerase Gamma Catalytic Subunit
TRAF6: Tumor Necrosis Factor Receptor-Associated Factor 6
SIRT1: Silent information regulator 1
ATP: Adenosine Triphosphate
MMP: Mitochondrial Membrane Potential
GSK-3β: Glycogen synthase kinase 3β
Tom20: Translocase of the outer mitochondrial membrane 20
MCL-1/BAK: myeloid cell leukemia-1/BCL-2 Antagonist 1
p53: Tumor Protein 53
K24: Lysine 24
CL: Cardiolipin
PA: phosphatidic acid
OMM: Outer Mitochondrial Membrane
IMM: Inner Membrane Membrane
NEDD4-1: Neural Precursor Cell Expressed, Developmentally Down-regulated Protein 4-1
MDM35: Mitochondrial Distribution and Morphology Protein 35
PINK1: PTEN induced putative kinase 1
Parkin: Parkin rbr E3 ub protein Ligase
GTPase: Guanosine Triphosphatase
Drp1: Dynamin-related protein 1
MFN1: Mitofusin 1
MFN2: Mitofusin 2
OPA1: Optic Atrophy 1
Fis1: Mitochondrial fission factor
Gβ2: G-protein β2
CCCP: Carbonyl cyanide 3-chlorophenylhydrazone
PGAM5: Phosphoglycerate mutase 5
CUL1: Cullin1
FBX: F-box protein
Fzo1: Fusion of Mitochondria Protein 1
6-OHDA: 6-hydroxydopamine
IKKγ: Inhibitor of Nuclear Factor Kappa-B Kinase Subunit Gamma
OMA1: Optic Atrophy 1
MUL1: Mitochondrial E3 ubiquitin protein ligase 1
PARL: Presenilin-Associated Rhomboid-Like
ULK1: Unc-51 Like Autophagy Activating Kinase 1
SIAH1: Seven in Absentia Homolog 1
LC3: Microtubule-associated protein 1 light chain 3
Atg: Autophagy-related
LAMP1: Lysosomal-associated membrane protein 1
SUMOylation: Small Ubiquitin-like Modifier Conjugation
HIF-1α: Hypoxia-inducible factor 1-alpha
ETC: Electron Transport Chain
H2Aub: histone H2A ubiquitination
Hsp27: heat shock protein 27
PFKP: Phosphofructokinase Platelet
HRD1: HMG-CoA Reductase Degradation 1
NADK: Nicotinamide Adenine Dinucleotide Kinase
STAT3: Signal Transducer and Activator of Transcription 3
mTORC1: mechanistic target of rapamycin complex 1
LAMTOR1: mTOR Activator 1
BDNF: brain-derived neurotrophic factor
VDAC3: Voltage dependent anion channel 3
CDK9: Cyclin-dependent kinase 9
TCM: traditional Chinese medicine
HBx: hepatitis B virus -encoded X protein
UBE2O: Ubiquitin conjugating enzyme E2 O
HMGCR: 3-hydroxy-3-methylglutaryl-CoA reductase
TACE: Transarterial chemoembolization
STOML2: Stomatin like 2
IKE: imidazole erastin
RBCK1: RANBP2-type and C3HC4-type zinc finger containing 1
MAVS: mitochondrial antiviral signaling
LA: Lipoic acid
NLRP3: Nucleotide-binding oligomerization domain, Leucine-rich repeat, and Pyrin domain-containing protein 3
RIPK1: Receptor-interacting protein kinase 1
MCU: mitochondrial calcium uniporter
PFK1: phosphofructokinase-1
GPC4: glypican 4
USP36: Ubiquitin specific peptidase 36
XRCC1: X-ray repair cross-complementing 1
SOX13: SRY-box transcription factor 13
AIF: apoptosis-inducing factor
SNPH: Syntaphilin
